# Oxidative stress and its association with cardiovascular disease in chronic renal failure patients

**DOI:** 10.4103/0971-4065.75218

**Published:** 2011

**Authors:** Z. Hambali, Z. Ahmad, S. Arab, H. Khazaai

**Affiliations:** 1 Department of Pathology, Chemical Pathology Unit, Faculty of Medicine and Health Sciences, Universiti Putra Malaysia, 43400 UPM Serdang, Selangor, Malaysia; 2 Institute of Bioscience, Molecular Biomedicine Laboratory, Universiti Putra Malaysia, 43400 UPM Serdang, Selangor, Malaysia; 3 Department of Biomedical Science, Faculty of Medicine and Health Sciences, Universiti Putra Malaysia, 43400 UPM Serdang, Selangor, Malaysia

**Keywords:** Chronic renal failure, CVD, oxidative stress

## Abstract

Cardiovascular disease (CVD) is responsible for the majority of deaths in chronic renal failure (CRF). Oxidative stress plays a key role in pathogenesis of atherosclerosis and CVD, which is promoted by the production of reactive oxygen species (ROS) and impaired antioxidant enzymes. These ROS react with nitric oxide (NO) to produce cytotoxic reactive nitrogen species that cause oxidative injury to the endothelium. This study evaluated biomarkers of oxidative stress, NOx (total NO_2_ and NO_3_), and superoxide dismutase (SOD) enzyme in normal control and CRF patients as case group and correlated their association with CVD. This cross sectional study involved 173 CRF patients on different modes of treatment (hemodialysis, continuous ambulatory peritoneal dialysis (CAPD), and predialysis). Of these, 74 had CVD. The control group consisted of 33 healthy subjects who had no history of CRF and CVD. Both NOx and SOD levels were significantly lower (*P*<0.05, *P*<0.001, respectively) in the case group. Comparing between CRF patients with and without CVD, SOD level was found to be significantly lower in CRF patients with CVD (*P*<0.05). Logistic regression analysis showed significant association of CVD event with age, male gender, diabetes, SOD level, and lipid profile in CRF patients. Oxidative stress occurs in the CRF patients with or without CVD. This study found that NOx and SOD levels were reduced in all CRF patients with or without CVD. However, it was noted that the levels of these biomarkers of oxidative stress were significantly lower in CRF patients with CVD compared with CRF patients without CVD. Therefore, these oxidative stress markers maybe contributing factors in the pathogenesis of CVD in patients with CRF.

## Introduction

Oxidative stress plays a central role in the pathogenesis of atherosclerosis that leads to cardiovascular complication in chronic renal failure (CRF) patients.[1] CRF patients experience a significantly increased rate of atherosclerotic complications. Thus, a higher prevalence of cardiovascular disease (CVD) is seen in CRF patients compared with other populations. Epidemiological studies have shown several risk factors for CVD in CRF patients, such as hypertension, diabetes, and hyperlipidemia, but nontraditional risk factors such as oxidative stress may also contribute to these complications.[2]

CRF is also associated with oxidative stress and its presence is evidenced by an imbalance between pro-and anti-oxidant systems. This association has been established in hemodialysis,[3,4] continuous ambulatory peritoneal dialysis (CAPD),[5] and predialysis patients with CRF.[5] Nitric oxide (NO) is a gaseous lipophilic free radical cellular messenger generated by three different isoforms of nitric oxide synthases (NOS), i.e., neuronal (nNOS), inducible (iNOS), and endothelial NOS (eNOS). NO is part of the antioxidant system and plays an important role in the protection against CVD. The cardioprotective roles of NO include regulation of blood pressure and vascular tone, inhibition of platelet aggregation and leukocyte adhesion, and prevention of smooth muscle cell proliferation. Highly reactive radicals and cytotoxic products such as peroxynitrite (ONOO) and peroxynitrous acid (ONOOH) were remarkably increased in an oxidative stress state.[6] These reactive oxygen species (ROS) react with NO, causing its inactivation.[7,8] These conditions resulted in deficiency of functional NO which can contribute to hypertension.[6,9] Many risk factors have been identified for CVD but their associations are not clear. This study was undertaken to examine nontraditional risk factors, especially indicators of oxidative stress in association with the development of CVD in CRF patients.

## Patients and Methods

Individuals with CRF with and without CVD from Serdang Hospital, Malaysia were recruited for this study. Patients with CRF were selected based on glomerular filtration rate for the classification of kidney disease.[10] Of 173 patients, 66 were in CKD stage 3-4 (predialysis) and 107 were CKD stage 5_D_ (dialysis) for this study. The selected population consisted of 87 men (50.2%) and 86 women (49.8%) aged between 18 to 80 years. Patients on dialysis were undergoing either hemodialysis or CAPD; clinically stable and free from active infections or autoimmune diseases were recruited for the study. None of these patients received immunosuppressive treatment or nonsteroidal antiinflammatory drugs at any time during the study. Seventy-four of these patients had clinical symptoms or a history of CVD (myocardial infarction, chronic ischaemic heart disease, atherosclerosis ischaemic heart disease, or congestive heart failure). The control group consisted of 33 healthy subjects between the age of 18 to 80 years, who had no history of CRF or CVD. The study was approved by the Ethics Committee of the Faculty of Medicine and Health Sciences of Universiti Putra Malaysia (UPM). Informed consent was obtained from all subjects before they participated in the study.

10 ml of blood was drawn into tubes containing ethylenediaminetetraacetic acid (EDTA) from each subject after on overnight fasting. The plasma was prepared and stored at -80°C until required for determination of biochemical markers. Lipid profile (HDL-C, LDL-C, total cholesterol, and triglyceride) concentrations were assayed by using homogeneous enzymatic colorimetric methods and analyzed by Hitachi 904 clinical chemistry autoanalyzer. Superoxide dismutase (SOD) levels were measured by using a RANSOD SOD kit from Randox, UK and analyzed using Selectra autoanalyzer. This method employed xanthine and xanthine oxidase to generate superoxide radicals which react with 2-(4-iodophenyl)-3-(4-nitrophenol)-5-phenyltetrazolium chloride (I.N.T.) to form a red formazan dye. Determination of nitrate and nitrite (NOx) was based on the Griess reaction in which a chromophore with a strong absorbance at 545 nm was formed by the reaction of nitrite with a mixture of N-naphthyl ethylenediamine and sulfanilamide. For NOx determination, aliquots of the sample were mixed with cadmium in glycine buffer at pH 9.7 to reduce nitrate to nitrite, which was then mixed with fresh reagent. The absorbance was measured by spectrophotometer (UV light- SECOMAN, France).

The results of this study were presented as means±SD for normally distributed variables and as median (range) for variables with a skewed distribution. The differences of normally distributed variables between 2 groups were assessed by t-test and analysis of variance (ANOVA), whereas variables with non-Gaussian distribution was compared by using nonparametric Mann-Whitney and Kruskal-Wallis tests, respectively. All *P* values were two-tailed and differences were considered significant if *P* values were ≤0.05. The association between the dependent and independent variables was determined by the correlation test. Association of risk factors for CVD outcome by using logistic regression analysis was performed between two groups of CRF, with and without CVD as the dependent variable.

## Results

Table 1 shows the demographic, clinical characteristic and oxidative stress parameters (SOD, NOx), and lipid profile of predialysis and dialysis patients. Age, BMI, and waist circumference values were significantly (*P*<0.05) higher in predialysis as compared with dialysis patients. The results of duration of CRF condition showed higher and significant differences (*P*<0.001) in dialysis cases than in predialysis. The prevalence of diabetes mellitus was significantly greater in predialysis than in dialysis patients (*P*<0.001). Compared with the controls, dialysis (*P*<0.001) and predialysis patients (*P*<0.05) showed a significant elevation in systolic blood pressure. It showed a significant elevation in dialysis as compared with predialysis patients (*P*<0.05). There was no significant difference in the use of antilipidemic drugs between two case groups. However, in use of hypoglycemic drugs, predialysis patients showed significantly higher consumption as compared with dialysis patients (*P*<0.05) as well as the use of antihypertension drugs in predialysis was more common, which was the significant difference between two groups (*P*<0.05). As illustrated in Table 1, both two case groups demonstrated similar significant (*P*<0.05) increased level of cholesterol and triglyceride in comparison with control.

**Table 1:** Demographic and clinical characteristics and biochemical parameters of predialysis and dialysis patients and control

Polymorphisms	Control	Chronic renal failure	
		Dialysis	Predialysis
Number of cases	33	107	66
Age (year)	47.3±16.5	64.5±13.4	57.4±15.6^a^
Gender: male/female	14/19	53/44	39/27
BMI (kg/cm^2^)	23.7±3.2	24.9±4.0	26.3±4.6^a^
Waist circumference (cm)	83.4±9.7	86.5±10.0	91.2±9.8^a^
Duration of CRF (month)	–	62.5±21.5	31.6±9.7^b^
Systolic BP (mmHg)	126.4±18.1	143.5±24.0^b^	138.6±27.0^a^,^c^
Diastolic BP (mmHg)	76.2±10.5	72.3±12.3	77.6±17.0^c^
Diabetes, n (%)	–	62 (58)	54 (81)^d^
Smoker, n (%)	3 (9)	19 (17.7)^b^	13 (19.6)^b^
Drugs			
Antilipidemic, n (%)	–	31 (29)	19 (29)
Hypoglycemic, n (%)	–	29 (27)	23 (35)^c^
Antihypertension, n (%)	–	35 (33)	31 (47)^c^
Oxidative stress parameters			
NOx (μmol/l)	30.31±13.20	21.80±13.40^b^	20.20±7.30^b^
SOD (U/ml)	6.52±0.33	5.20±0.72^b^	5.42±0.40^b^
Lipid profile			
Cholesterol (mg/dl)	175.55±20.67	199.68±42.90^a^	218.40±42.91^a^
LDL (mg/dl)	102.57±20.72	117.10±42.12	119.12±38.10
HDL (mg/dl)	51.87±13.66	39.00±14.43	42.91±15.60
Triglyceride (mg/dl)	114.45±42.82	226.33±80.60^b^	252.70±96.12^b^

Data were presented using ANOVA post test (Tukey test) and t test

a *P*<0.05

b *P*<0.001 patients vs control

c *P*< 0.05

d *P*<0.001 predialysis vs dialysis

CRF = Chronic renal failure; BMI = Body mass index; BP = Blood pressure; NOx = total (NO_2_ + NO_3_); SOD = Superoxide dismutase; LDL = Low-density lipoprotein; HDL = High-density lipoprotein

Moreover, there were no statistically significant differences in lipid profile between those two case groups. Despite these, SOD level was significantly (*P*<0.05) lower in predialysis and dialysis patients when compared with the control. NO concentration was not different in both groups (predialysis and dialysis). NO, SOD, and creatinine levels were also analyzed in CRF patients with and without CVD, regardless of whether these patients were on dialysis. Those who were on dialysis were further classified into CAPD and hemodialysis groups [Table 2]. NO levels were significantly lower in CRF patients with CVD (*P*<0.001) and without CVD (*P*<0.05) in both CAPD group as well as predialysis group when compared with the control group. CAPD with CVD had a significant decrease in the levels of NO and SOD in comparison with CAPD without CVD (*P*<0.05). However, no significant difference was found in the CRF patients with or without CVD who were on hemodialysis when compared with control group. Significant lowering of SOD levels were detected in all groups of CRF patients when compared with control group (*P*<0.001). Compared with hemodialysis with CVD, hemodialysis without CVD showed a significant increase in the level of creatinine and decrease in the level of triglyceride (*P*<0.05). In general, all CRF patients had higher levels of cholesterol, LDL, and triglyceride as compared with control group. Pearson correlation analysis showed that NOx concentration was negatively correlated with lipid profile and positively with age, creatinine and SOD levels. SOD enzyme level was negatively correlated with all parameters except NOx and HDL. Logistic regression analysis was performed among all CRF patients (predialysis and dialysis) who were categorized in two groups of CRF patients, with and without CVD as the dependent variable [Table 3]. All anthropometric, physiological, and biochemical variables were entered; however, only the variables age (odds ratio [OR] = 1.003, *P*<0.001) and lipid profile showed strong association in adjusted model between these variables and occurrence of CVD, but male gender (OR=1.05, *P*<0.05), diabetes (OR=2.342, *P*<0.01), and SOD levels (OR=1.702, *P*<0.05) showed a significant association in crude model.

**Table 2:** Biochemical parameters of chronic renal failure patients with different modes of treatment *vs* the control

	Control	Pre-dialysis		Dialysis			
				CAPD		Hemodialysis	
		With CVD	Without CVD	With CVD	Without CVD	With CVD	Without CVD
Number	33	33	33	19	33	22	33
Biochemical parameters							
Creatinine (mg/dl)	0.72±0.44	3.12±1.46^b^	3.10±2.42^b^	7.80±3.04^b^	8.25±2.92^b^	7.18±2.11^b^	8.54±2.36^b^,^d^
NOx (μmol/l)	30.3±13.2	19.9±9.6^b^	21.4±5.4^a^	13.8±12.4^b^	21.2±15.5^a^,^c^	25.4±12.2	25.3±10.2
SOD (U/ml)	6.50±0.33	5.42±0.37^b^	5.42±0.42^b^	5.20±0.56^b^	5.40±0.64^b^,^c^	5.32±0.85^b^	5.12±0.58^b^
Cholesterol (mg/dl)	175.55±20.67	210.60±20.34^a^	219.18±47.58^a^	214.16±55.38^b^	208.16±51.38^a^	187.23±24.96	180.57±31.21
LDL-c (mg/dl)	102.57±20.72	113.12±31.98	120.90±39.00	124.80±43.68	132.60±46.81^a^	109.98±21.06	102.18±33.54
HDL-c (mg/dl)	51.87±13.66	42.92±14.04	43.68±17.16	39.00±14.82	43.68±15.21	39.10±12.48	39.08±10.14
Triglyceride (mg/dl)	114.45±42.82	232.48±54.88^a^	220.65±113.86^a^	260.85±116.44^b^	238.26±70.57^a^	200.98±77.31^a^	196.98±106.31^a^

Data were given as mean±SD and the significant differences between groups were assessed by ANOVA post test (Tukey test)

a *P*<0.05

b *P*<0.001 patients vs controls

c *P*<0.05 CAPD with CVD vs CAPD without CVD

d *P*<0.05 hemodialysis with CVD vs hemodialysis without CVD.NOx = total (NO_2_ + NO_3_);

SOD = Superoxide dismutase; CAPD = Continuous ambulatory peritoneal dialysis; CVD = Cardiovascular disease; LDL = Low-density lipoprotein; HDL = High-density lipoprotein

**Table 3:** Logistic regression analysis, crude and adjusted odds ratio, and 95% confidence intervals for the chronic renal failure patients

Variables	OR crude			OR adjusted		
	*P* value	Exp (ß)	95% Cl	*P* value	Exp (ß)	95% Cl
Age	0.001^*^	1.005	1.002-1.006	0.003^*^	1.003	1.000-1.005
Gender/male	0.050^*^	1.050	1.005-1.044	0.716	0.859	0.378-1.950
Diabetes	0.013^*^	2.343	1.195-8.037	0.296	0.639	0.276-1.481
SOD	0.044^*^	1.702	1.015-2.852	0.920	1.706	1.014-3.177
Cholesterol	0.870	1.005	0.742-1.287	0.020^*^	1.211	1.180-1.305
Triglyceride	0.050^*^	1.056	1.005-1.806	0.040^*^	1.595	1.012-2.514
LDL-c	0.648	1.075	0.788-1.467	0.050^*^	2.418	1.000-5.875

The odds ratios for continuous variables are presented as the standardized regression coefficients as the term Exp (ß). Adjusted model = compared with other variables; Crude model - single or no adjusted with other variables; CRF = Chronic renal failure; SOD = Superoxide dismutase; OR = Odds ratio; CI = Confidence interval

## Discussion

The aim of the present study was to evaluate oxidative stress biomarkers as measured by plasma levels of NOx and SOD enzyme among CRF patients with and without CVD on different modes of treatment. A further purpose was to identify the relationship between these studied parameters with CVD in these patients. The results in this study showed that CRF patients with CVD were usually older than CRF without CVD. The role of age in prevalence of CVD events in CRF patients was confirmed by result of logistic regression analysis and also a study by Hemmelgarn *et al*.[8] who reported that renal insufficiency is a marker for elevated CVD risk in a community of elderly adults. In the present investigation, the levels of NOx and SOD in CRF patients were significantly lower than healthy control group. According to previous studies,[6,11] the reasons for lower NOx and SOD levels in CRF patients may relate to excess production of ROS due to oxidative stress in plasma and red blood cells in CRF patients. Similar result of SOD enzyme activity was shown previously by Sasikala *et al*.[12] CRF patients with CVD showed significantly lower activity of SOD enzyme in comparison with CRF patients without CVD. Our results showed significant association between SOD levels and occurrence of CVD in CRF patients. Data from several studies have shown that the development and progression of atherosclerotic disease in animal models and human is highly due to the imbalance of oxidant and antioxidant enzymes.[13] SOD level had significant and negative correlation with age, creatinine, cholesterol, and triglyceride. Moreover, our results presented here show strong association between levels of triglyceride, LDL, and cholesterol with the occurrence of CVD. These results which proved to be agreeable with other investigators’ results revealed that classic risk factors of atherosclerosis, such as age, duration of CRF condition, and elevated lipid profile, contribute to the development of carotid atherosclerosis in patients with CRF.[14] The function of NO in CRF patients on dialysis is important in this report. NO can play both a protective and a toxic role in the cell.[9] In our study, we showed that stable-end NO metabolite plasma levels (nitrites and nitrates) were decreased in all CRF patients when compared with control group. These results had significant differences in most groups, except hemodialysis, suggesting that long-term hemodialysis are associated with chronic stimulation of NO production.[15] Endothelial dysfunction is an early event in the development of atherosclerosis in CRF patients[16,17] and is known to cause gathering of atherosclerotic plaques.[18] It is observed that progression in endothelial dysfunction depends on the degree of oxidative stress.[19,20] Atherosclerosis manifests more in adult CRF patients[21] and may be one of the causes of defect in NO production, because a compact expression of eNOS enzyme has recently been reported to occur in conditions of atherosclerosis.[22] The endothelial function may be impaired by risk factors for CVD such as hypertension, hyperlipidemia, and diabetes.

On the other hand, impaired NO production may also have resulted from increasing endogenous NOS-inhibitor, asymmetric dimethylarginine (ADMA) in patients with end-stage renal disease, which is well documented in previous studies.[17,23] However, one of the limitations in this study was the inability to separate the various isoforms of NOS (endothelial, neuronal, and inducible). Both renal failure and CVD have been reported in animal studies and were associated with decreased expression of iNOS which has direct association with the stage of disease.[22–25] On the other hand, the reduction in NO production may also relate to the atherosclerosis process. Recent studies indicated that iNOS expression is important in prevention of neointima proliferation and endothelial regeneration.[26,27] The present results indicate multiple interactions between plasma SOD, NOx, lipid profile, male gender, age, and diabetes, with the occurrence of CVD in CRF patients. It is postulated that low SOD level implicates the reduction of NO production which is due to oxidation/inactivation of NO by ROS. The severity of CRF complications is highly correlated with the oxidative stress status of the patient, which may lead to the pathogenesis of CVD in patients with CRF. Further studies are needed to determine with certainty the degree of risk of CVD associated with changes in these oxidative stress markers and in CRF patients.

## References

[1] Locatelli F, Bommer J, London GM, Martín-Malo A, Wanner C, Yaqoob M (2005). Cardiovascular disease determinants in chronic renal failure: clinical approach and treatment. Nephrol Dial Transplant.

[2] Stenvinkel P, Pecoits-Filho R, Lindholm B (2003). Coronary artery disease in end-stage renal disease: no longer a simple plumbing problem. J Am Soc Nephrol.

[3] Mandal KA, Woodi M, Sood V, Krishnaswamy RP, Rao A, Ballal S (2007). Quantitation and characterization of glutathionyl haemoglobin as an oxidative stress marker in chronic renal failure by mass spectrometry. Clin Biochem.

[4] Brunini TM, Mendes-Ribeiro AC, Ellory JC, Mann GE (2007). Platelet nitric oxide synthesis in uremia and malnutrition: A role for L-arginine supplementation in vascular protection?. Cardiovasc Res.

[5] Ronco C, Brendolan A, Levin NW (2005). Cardiovascular Disorders in Hemodialysis. 14th International Course on Hemodialysis, Vicenza, May.

[6] Vaziri ND, Wang XQ, Oveisi F, Rad B (2000). Induction of oxidative stress by glutathione depletion causes severe hypertension in normal rats. Hypertension.

[7] Annuk M, Zilmer M, Lind L, Linde T, Fellström B (2001). Oxidative stress and endothelial function in chronic renal failure. J Am Soc Nephrol.

[8] Hemmelgarn BR, Manns BJ, Zhang J, Tonelli M, Klarenbach S, Walsh M (2007). Association between multidisciplinary care and survival for elderly patients with chronic kidney disease. J Am Soc Nephrol.

[9] Vaziri ND, Ni Z, Oveisi F, Liang K, Pandian R (2002). Enhanced nitric oxide inactivation and protein nitration by reactive oxygen species in renal insufficiency. Hypertension.

[10] Levey AS, Coresh J, Bolton K (2002). Disease outcome quality initiative. K/DOQI clinical practice guidelines for chronic kidney disease evaluation, classification, and stratification. Kidney Am J Kidney Dis.

[11] Vaziri ND (2002). Oxidative stress in uremia: nature, mechanisms, and potential consequences. Semin Nephrol.

[12] Sasikala M, Sadasivudu B, Subramanyam C (2000). A putative role for calcineurin in lymphopenia associated with chronic renal failure. Clin Biochem.

[13] Wassmann S, Wassmann K, Nickenig G (2004). Modulation of oxidant and antioxidant enzyme expression and function in vascular cells. Hypertension.

[14] Shoji T, Ishimura E, Inaba M, Tabata T, Nishizawa Y (2001). Atherogenic lipoproteins in end-stage renal disease. Am J Kidney Dis.

[15] Clermont G, Lecour S, Lahet J, Siohan P (2000). Alteration in plasma antioxidant Capacities in chronic renal failure and haemodialysis patients. Cardiovasc Res.

[16] Choi JH, Kim KL, Huh W, Kim B, Byun J, Suh W (2004). Decreased number and impaired angiogenic function of endothelial progenitor cells in patients with chronic renal failure. Arterioscler Thromb Vasc Biol.

[17] Wong JR, Wu (2008). Endothelial dysfunction and chronic kidney disease: treatment options. Curr Opin Investig Drugs.

[18] Cross J (2002). Endothelial dysfunction in uraemia. Blood Purif.

[19] Matsuoka H (2001). Endothelial dysfunction associated with oxidative stress in human. Diabetes Res and Clin Practice.

[20] Heitzer T, Schlinzig T, Krohn K, Meinertz T, Munzel T (2001). Endothelial dysfunction, oxidative stress, and risk of cardiovascular events in patients with coronary artery disease. Circulation.

[21] London GM, Drueke TB (1997). Atherosclerosis and arteriosclerosis in chronic renal failure. Kidney Int.

[22] Wilcoc JN, Subramanian RR, Tracey WR, Pollock JS, Harrison DG, Marsden PA (1997). Expression of multiple isoforms of nitric oxide synthase in normal and atherosclerotic. Arterioscler Thromb Vasc Biol.

[23] van Guldener C, Nanayakkara PW, Stehouwer CD (2007). Homocysteine and asymmetric dimethylarginine (ADMA): biochemically linked but differently related to vascular disease in chronic kidney disease. Clin Chem Lab Med.

[24] Vaziri ND, Zhenmin NI, Wang XQ, Oveisi F, Zhou XJ (1998). Down regulation of nitric oxide synthase in chronic renal insufficiency: role of excess PTH. Am J Physiol.

[25] Aiello S, Noris M, Todeschini M, Zappella S, Foglieni C, Benigni A (1998). Renal and systemic nitric oxide synthesis in rats with renal mass reduction. Kidney Int.

[26] Kawashima S, Yokoyama M (2004). Dysfunction of endothelial nitric oxide synthase and atherosclerosis. Arterioscler Thromb Vasc Biol.

[27] Yan Z, Hansson GK (1998). Over expression of inducible nitric oxide synthase by neointimal smooth muscle cells. Circ Res.

